# Students and Maternity Departments

**Published:** 1896-08-29

**Authors:** 


### The Hospital

AN INSTITUTIONAL JOURNAL OF

The Medical Sciences and Hospital Administration,

WITH

AN EXTRA NURSING SUPPLEMENT.

VOL. XX.—No. 518.] [AUG. 29, 1896.

### STUDENTS AND MATERNITY DEPARTMENTS.

THE report of an inquest recently held in Lambeth draws attention to a weak spot in the management of some of our largest hospitals which demands the immediate attention of those responsible in the matter. In this particular case it was in connection with St. Thomas's Hospital that the difficulty arose, but we have no reason to believe that this hospital is any more in fault than many others. The father of the child which died is reported to have stated that his wife had made arrangements to be attended in her confinement by someone from the maternity department at St. Thomas's Hospital. On going for the doctor he had to wait an hour before he could see him, and on returning home he found that the child was born. The accoucheur attended shortly after, and promised to come again later in the day, but he did not call until the evening of the next day, and then the child was dead. The obstetric clerk gave as his reason for not returning to the house sooner the fact that he was exceedingly busy, and the junior obstetric house physician stated that they had only three obstetric clerks at the hospital.

The case is a simple one of an over-worked maternity department, and it is very probable, as was stated in the evidence, that if twenty medical men had been in attendance they could not have saved the child's life. Nevertheless, it raises again the question, which is continually cropping up as to the conditions under which the home patients are attended by students connected with the maternity departments of large school hospitals.

That students must have opportunities for attending midwifery before they receive their qualifications goes without saying. The question is as to the conditions under which the necessary experience should be gained, and we have no hesitation in saying that at many hospitals these conditions are the reverse of favourable. Students who attend maternity cases must have passed their second professional examination, and have attended courses of lectures on midwifery and practical obstetrics. In the theory of their work then they ought to be well up. But as regards practical teaching at the bedside by responsible medical officers, we fear it is of the smallest. With their heads full of theory they are turned adrift to pick up practical experience, often in the homes of the very poorest, amid surroundings such as would make even the most experienced almost despair of satisfactory results. Surrounded by dirt of every sort, and worried to exasperation by the exuberant insect life, without any of the ordinary appliances common in decent homes, may be without even a basin in which to wash his hands, an unfledged student is asked to do aseptic midwifery! It would puzzle a wiser head than his; and it speaks well for the pluck and adaptability of our medical students, and for their earnest desire to do well by the poor women entrusted to their care that accidents do not occur more often than they do.

What is certain is that amid such surroundings the student gains from each case the minimum of experience with the maximum of trouble and discomfort, and that although he gains practice and gets his certificate, the teaching he receives is a *quantité négligeable* so long as his cases pursue an ordinary course.

If, then, this sort of practice is the best that can be offered for the learning of midwifery in London, at least it should be so arranged that its difficulties should not be aggravated by the work being done under the stress of hurry by students exhausted by over work. But this is exactly the state of affairs which is encouraged by too many hospitals. At the inquest alluded to it was stated that there were only three obstetric clerks. Now. on turning to the prospectus of the medical school for 1895 we find that 1,912 confinements were attended during the year, which amounts to over thirty-six a week, or twelve a week for each clerk. This may not be very excessive, although, if we consider the after visiting and the much longer time which an inexperienced man ought to devote to each case than is necessary for those who have had large practice in the art, we think these young men will find it by no means easy work if it is to be properly done. In comparison with this we may take St. Bartholomew's, where, with a very much larger number of students, there is a smaller maternity; and as an extreme example in the opposite direction, University College Hospital, where, with a still smaller number of students for the full curriculum, the maternity is far larger than at either of the others, 2,591 cases having been attended in the year.

It is quite easy to appreciate the motives which lead obstetric physicians to encourage large maternities, but we cannot but think that to the student personal teaching and the demonstration of the points in each case as they arise, even though the cases might be fewer in number, would be of more help than the present system; and as to the patients, we are sure that to hand them over to hurried and exhausted students is wrong.

